# KRIT1 in vascular biology and beyond

**DOI:** 10.1042/BSR20231675

**Published:** 2024-07-19

**Authors:** Angela J. Glading

**Affiliations:** Department of Pharmacology and Physiology, University of Rochester, Rochester, NY, U.S.A.

**Keywords:** CCM, KRIT1, tumor suppressor, vascular malformations

## Abstract

KRIT1 is a 75 kDa scaffolding protein which regulates endothelial cell phenotype by limiting the response to inflammatory stimuli and maintaining a quiescent and stable endothelial barrier. Loss-of-function mutations in KRIT1 lead to the development of cerebral cavernous malformations (CCM), a disease marked by the formation of abnormal blood vessels which exhibit a loss of barrier function, increased endothelial proliferation, and altered gene expression. While many advances have been made in our understanding of how KRIT1, and the functionally related proteins CCM2 and PDCD10, contribute to the regulation of blood vessels and the vascular barrier, some important open questions remain. In addition, KRIT1 is widely expressed and KRIT1 and the other CCM proteins have been shown to play important roles in non-endothelial cell types and tissues, which may or may not be related to their role as pathogenic originators of CCM. In this review, we discuss some of the unsettled questions regarding the role of KRIT1 in vascular physiology and discuss recent advances that suggest this ubiquitously expressed protein may have a role beyond the endothelial cell.

## Introduction

The first publication describing KRIT1 was published in 1997, when it was identified as an interactor of the Ras family small GTPase, Rap1a, by yeast two-hybrid. Subsequently named Krev1/Rap1a Interaction Trapped, *Krit1* was mapped initially to chromosome 7q21-22 [1], where a gene mutated in Cerebral Cavernous Malformations (CCM) had recently been mapped [2,3]. This gene locus, narrowed to 7q21.2, was initially thought to encode 12 exons generating a 58 kDa protein; however, cloning of the gene revealed four additional 5′ coding exons [4], bringing the total length of KRIT1 to 736 amino acids with a molecular weight of 75kDa. Subsequent studies have demonstrated that loss-of-function mutations in *Krit1* are found in multiple families worldwide affected by CCM, making it one of three genes associated with the hereditary form of CCM [5]. Indeed, in the United States, mutations in *Krit1* are the leading cause of CCM, due in part to the high prevalence of a specific founder mutation in the population of the Southwest [2,3].

In the more than two decades since the initial mapping of KRIT1 as a CCM-causing gene, significant progress has been made in understanding why loss of this protein leads to the abnormal vessel development and hemorrhage that characterize CCM. However, there are still many questions regarding KRIT1 and CCM disease that remain unanswered. In this review, we will discuss what is known about the role of KRIT1 in vascular biology, focusing on some of the remaining open questions, and discuss recent advances that suggest this ubiquitously expressed protein may have a role beyond the endothelial cell.

## KRIT1 in vascular biology

### The endothelium

**Figure 1:**
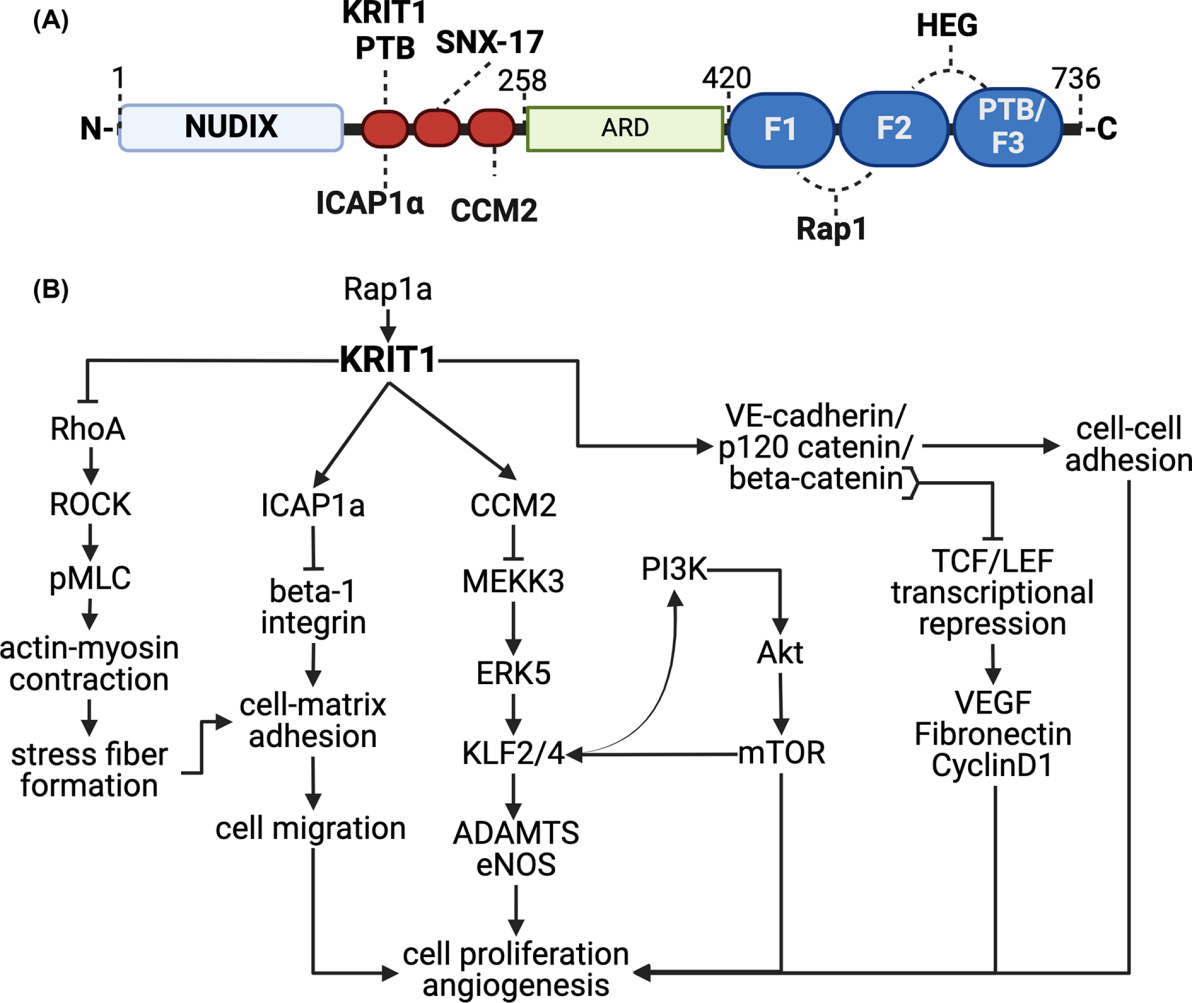
Domain structure of KRIT1 and signaling diagram of KRIT1-regulated pathways (**A**) KRIT1 contains several domains that mediate protein-protein interactions. The Nudix domain lacks the catalytic sequence which confers nucleotide hydrolase activity on other Nudix proteins. The NPXY motifs (red ovals) bind to phosphotyrosine binding domains (PTBs) in KRIT1, ICAP1α, sorting nexin-17 (SNX-17) and CCM2. Four ankyrin repeats can be found in the ankyrin repeat domain (ARD). The C-terminus contains a Band 4.1, ezrin, radixin, and moesin (FERM) domain. Surfaces on the FERM domain mediate binding to the GTPase Rap1a, and the orphan receptor Heart of Glass (HEG). (**B**) KRIT1 negatively regulates several pathways that govern endothelial cell proliferation, migration, and angiogenesis, and is critical for maintaining endothelial quiescence. Created with Biorender.com

KRIT1 contains several domains and amino acid motifs that mediate binding to other proteins and lacks functional enzymatic or nucleotide binding regions (Figure 1A); thus, KRIT1 is classified as a scaffolding protein, as are the functionally related proteins CCM2 and PDCD10. The domain structure of KRIT1 bears similarities to other scaffolding proteins which regulate integrin adhesion and the actin cytoskeleton, including talin, ezrin, radixin, and moesin, all members of the family of FERM domain containing proteins [2]. Unlike these other members of this family; however, KRIT1 lacks an actin binding domain. The C-terminal FERM domain of KRIT1 is divided into three globular subdomains (F1, F2, F3). Rap1, a small GTPase which regulates several cellular functions including cell adhesion [6], binds to an extended surface on the F1 and F2 lobes [7,8]. The orphan receptor Heart of Glass (HEG) binds to an interface between the F1 and F3 subdomains and may promote membrane localization of KRIT1 [9]. Interestingly, no ligands for the F3 subdomain, which encodes a phosphotyrosine binding domain (PTB), have been discovered other than KRIT1 itself, as the PTB mediates binding of the KRIT1 C-terminus to a NPXY motif (N^192^PAY) in the N-terminus [10]. This NPXY motif (N^192^PAY) is one of three such motifs in the N-terminal half of KRIT1, all of which are critical for important binding interactions. In addition to interacting with the KRIT1 PTB domain, N^192^PAY is also a ligand for the PTB domain of integrin cytoplasmic domain-associated protein-1α (ICAP1α), which can compete for binding with the KRIT1 PTB [10]. ICAP1α also competes with talin to bind to a NPXY motif on the cytoplasmic tail of β1 integrin, thus inhibiting talin-mediated β1 integrin activation [11,12]. KRIT1 has been shown to regulate ICAP1α in two ways. First, it can bind ICAP1α and prevent its interaction with β1 integrin, ostensibly increasing β1 integrin activity [13]. Second, KRIT1 binding regulates the stability of ICAP1α, as mutating the N^192^PAY sequence causes a proteosome-dependent reduction in ICAP1α protein level [14,15]. The second and third (according to sequence order) NPXY motifs in the N-terminal half of KRIT1 have been shown to mediate binding to sorting nexin-17 (SNX17) [16] and CCM2 [17], respectively. The functional consequence of binding to SNX-17 is unknown, though this protein has been suggested to regulate integrin recycling [18,19]. The interaction with CCM2, however, is critical for the function of the KRIT1/CCM2/PDCD10 complex, as a point mutation disrupting this interaction has been shown to cause CCM [17,20]. Other domains identified in the N-terminal half of KRIT1 include a Nudix (Nucleotide Diphosphate linked to an X moiety)-like domain (ama 1-170) [21] and four ankyrin repeats (ama 259-419) [22]. The functional role(s) of these domains remains unknown. The Nudix-like domain lacks the Nudix hydrolase catalytic domain but could retain nucleotide binding capabilities. The ankyrin repeats stack together to form an integrated domain that interacts with the F1 lobe of the FERM domain [22], and which may also form a binding surface for other protein–protein interactions.

Exactly how loss-of-function mutations in *Krit1* lead to the development of CCM has been an area of intense investigation for more than 20 years. The critical early discovery that a disrupted endothelial blood–brain barrier is present in CCM [23] led researchers to focus their attention on the role of KRIT1 in endothelial cells. Initially, KRIT1 was found to be localized to endothelial cell–cell contacts, as well as being present in the cytosol and nucleus [24]. Knockdown of KRIT1 decreased the stability of adherens junction complexes and led to increased endothelial permeability [24]. The destabilization of adherens junctions also up-regulated β-catenin dependent transcription and induced changes in gene expression [25], including up-regulation of *vegfa* and subsequent activation of VEGFR2 [26] (Figure 1B). In addition, loss of KRIT1 expression increased RhoA/Rho-associated protein kinase (ROCK) signaling to trigger cytoskeletal remodeling [24,27]. Loss of KRIT1 also stimulated MEKK3 activity that, via a MEK5-ERK5-MEK2 kinase cascade, led to increased expression of the transcription factors KLF2 and KLF4, to promote endothelial-to-mesenchymal transition (EndMT) [28] (Figure 1B). The development of an endothelial-specific, temporally-inducible *Krit1* knockout mouse model which recapitulates the human disease [29,30] bolstered these discoveries and revealed several potential therapeutic targets that could be inhibited to limit CCM-like lesion formation, including ROCK [31], VEGFR2 [32], and ERK5 [33]. In addition, in vivo studies showed that constitutively active *PIK3CA* (phosphatidylinositol-3 kinase, PI3K) could promote lesion formation [34], suggesting that inhibiting this enzyme could also be therapeutically advantageous.

Our understanding of the function of KRIT1 is driven primarily by what occurs in its absence, which implies that KRIT1 somehow limits the activation of these pathways. Unfortunately, the specific molecular interactions responsible remain unclear. For example, evidence suggests CCM2 binds to MEKK3 and blocks its activation [33,35]. How loss of KRIT1 would affect this interaction has not been determined, although it has been proposed that KRIT1 promotes CCM2 protein stability [14]. Similarly, the molecular mechanism by which KRIT1 and CCM2 limit RhoA/ROCK activation has not been defined. This lack of mechanistic detail limits our ability to understand and predict the consequences of blocking specific pathways, and likely our ability to design effective therapeutics. Notably, pharmacologic inhibition of these pathways has not been able to reduce lesion formation by more than ∼50% in preclinical studies; thus, more work is clearly needed to derive an integrated model of KRIT1 signaling in endothelial cells.

### Beyond the endothelium: the neurovascular unit

**Figure 2:**
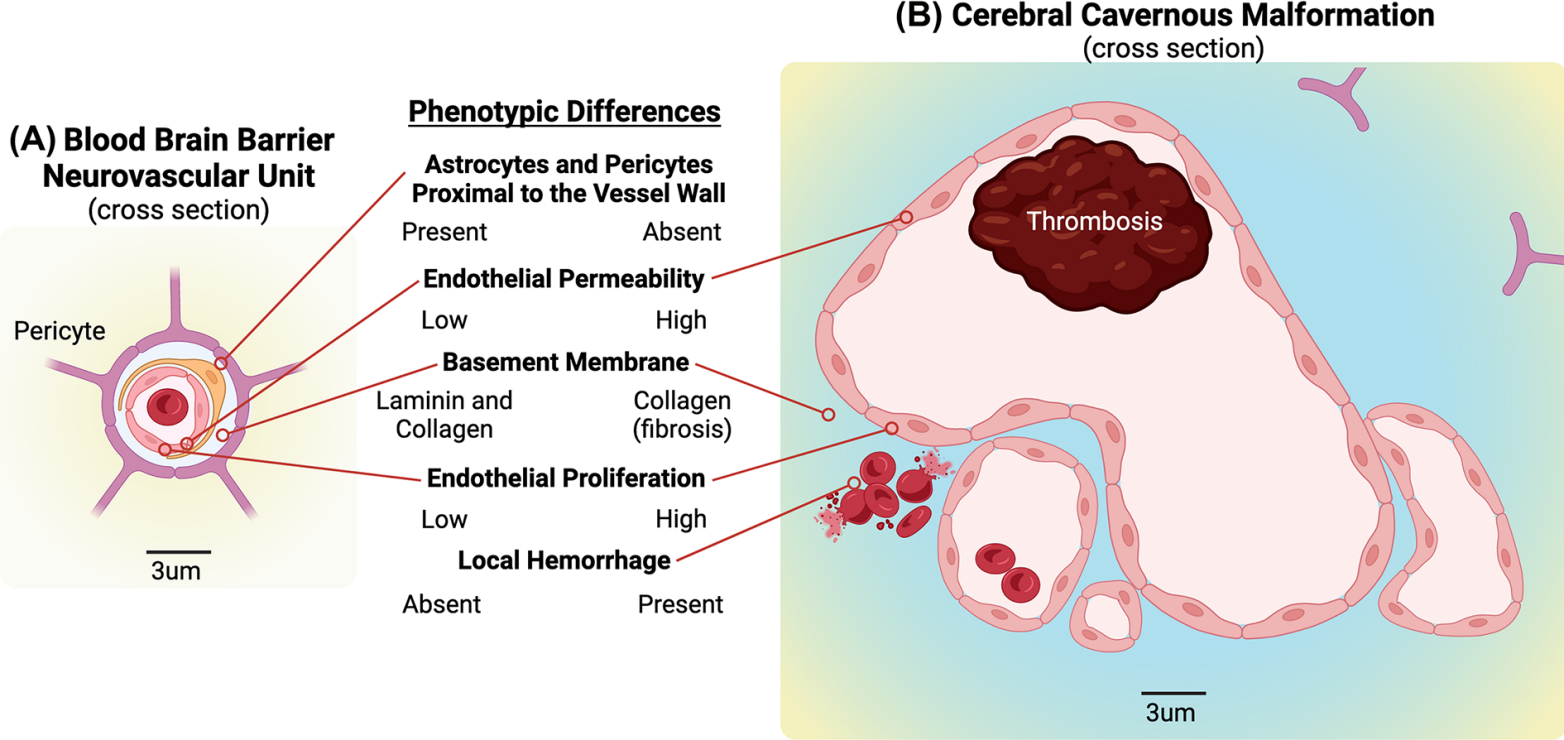
Comparison of structure of the normal blood-brain-barrier to the structure of a CCM lesion (**A**) The normal BBB comprises tight junction containing endothelial cells supported by adjacent pericytes and astrocytes. (**B**) In human CCM, the tight junctions are lost (disrupted cell–cell contacts) causing increased endothelial permeability, and pericytes and astrocytes are no longer immediately adjacent to the endothelium. In addition, changes in the local extracellular matrix and increased endothelial proliferation are observed. Created with Biorender.com.

As mentioned above, most of the research into the function of KRIT1 has focused on its role in endothelial cells and has been performed in cell culture models and endothelial-specific knockout animal models. However, blood vessels, even the simplest capillaries, are not just tubes of endothelial cells, but a complex tissue environment where both biochemical and mechanical signals act to control vessel development and endothelial phenotype. In the brain, where CCM lesions predominantly form, a specialized vascular barrier (blood-brain barrier, BBB) compartmentalizes the brain from circulating blood and maintains an optimal microenvironment for brain function [36]. Endothelial cells are the primary functional component of the BBB; however, several studies have demonstrated that the establishment of the BBB is not intrinsic to CNS endothelial cells, but derives from the local microenvironment, termed the neurovascular unit [37,38] (Figure 2A). While the identities of the specific BBB-inducing signals remain somewhat elusive, it is known that inputs from neurons, astrocytes, microglia, pericytes and the local extracellular matrix all contribute [36].

Multiple studies at both the mRNA and protein level indicate that KRIT1 is ubiquitously expressed across tissues and cell types [25,39,40], as are CCM2 and PDCD10 [41]. In hereditary CCM patients, homozygous mutant cells form lesions within a heterozygous tissue [42]; this haploinsufficiency could affect the function of non-endothelial cells in the lesion tissue environment [43]. Unfortunately, many of the published animal studies have been designed to examine the phenotype and function of homozygous null endothelial cells in a wild-type background. Even studies designed to assess the role of astrocytes, which are a critical component of the vascular microenvironment in the brain and contribute to the BBB [44], examined the effect of *PDCD10* or *KRIT1*-depletion in endothelial cells on wild-type astrocytes [45]. Nevertheless, these studies found that in *PDCD10* or *KRIT1* endothelial knockout animals, CCM formation was associated with areas of strong glial fibrillary acidic protein staining and immune cell infiltration, which may indicate astrocyte hypertrophy and reactive astrocytosis. Most notably, depletion of astrocytes in *PDCD10* knockout animals reduced lesion formation, supporting a role for these cells in CCM lesion formation [45] and suggesting that other cellular components of the BBB can contribute to CCM pathogenesis. Thus, the local microenvironment may be a critical component of the pathogenesis of CCM that has been largely overlooked.

### Beyond the endothelium: vessel morphology

Another clue that the local tissue microenvironment could be important to CCM pathogenesis is the fact that among the several types of vascular malformation, CCMs develop a unique pathological morphology. This morphology is characterized by enlarged, multiple-lumen containing, ‘cavernous’ vessels lined with a single layer of endothelium. These vessels are often surrounded by a thickened matrix layer, which replaces the basement membrane of normal vessels, and which is enriched in collagens and depleted of laminins [46,47] (Figure 2B). This contrasts with the morphological changes seen in other vascular malformations, such as arteriovenous malformations (AVMs) and hereditary hemorrhagic telangiectasias (HHT), which are marked by overgrowth of vessels that retain a tubular structure and a relatively normal basement membrane. In addition, these conditions do not exhibit the same tissue specificity of CCM [48]. This is a particularly interesting divergence given that many of the pathways found to be dysregulated in CCM are also dysregulated in other types of vascular malformation. For example, TGF-β signaling, a driver of EndMT, is up-regulated in both HHT [49,50] and CCM [28,51]. MAP kinase signaling (ERK, p38, JNK) is up-regulated in vascular RASopathies [52], infantile hemangioma [53], capillary malformation-arteriovenous malformation syndrome (CM-AVM) [54], Parkes-Weber syndrome (PWS) [55–57], Sturge-Weber syndrome (SWS) [58], HHT [59], and CCM [33]. Finally, PI3K signaling has recently been identified as an important player in CCM [34] but also contributes to PWS [57], SWS [58], venous malformation (VM and VMCM) [60,61], Klippel-Trénaunay syndrome (KTS) [62–64], HHT [65], and multiple *PIK3CA* syndromes which have associated vascular anomalies [66,67].

As the genetic alterations that cause the dysregulation of these pathways in each type of malformation can vary, both between and within each class, it is curious that the downstream signaling appears so conserved. The convergence of these signaling pathways and the processes they control (i.e. endothelial proliferation and migration) point to their fundamental role in regulating endothelial behavior and angiogenesis. However, this commonality confuses our ability to explain differences in affected vessel type, anatomical location, timing of disease development, malformation morphology, etc. that vary widely between these diseases. PWS consists of multiple, fast-flow, microscopic arteriovenous connections with variable capillary involvement in an enlarged limb, usually the lower extremity [68,69]. KTS is a slow flow combined vascular disorder involving abnormal capillaries, lymphatics, and veins [70,71]. Both infantile hemangioma [72] and SWS [68] vascular lesions consist of disorganized capillary-like vessels composed of immature endothelial cells and abundant α-smooth muscle actin positive perivascular cells. CCMs, on the other hand, are slow-flow capillary/venous malformations which lack perivascular cells [23,73]. Thus, there is currently no obvious correlation between slow- vs. fast-flow lesions or anatomical location and specific signaling pathways.

It is likely that heterogeneity in the effected endothelial cell population, developmental timing, and differences in the local vascular microenvironment contribute to these phenotypic differences in unique and unknown ways. In addition, there may be mechanistic differences in how these pathways are dysregulated downstream of the genetic defect. For example, in Sturge-Weber syndrome, MAP kinase activation occurs in stages, with JNK and ERK activated first, followed by AKT and PI3K which control lesion progression [74,75]. Genetic causes of SWS include mutations in *RASA1* (p120RasGAP) [76,77], *GNAQ* [78–80], and *PIK3CA* (PI3K) [68]. This progressive change in signaling may also be found in other types of malformations [81–84]; however, studies examining signaling changes during lesion development have not been performed in all types of vascular malformations, and thus any potential differences or similarities remain unknown. By teasing out the differences between the phenotypes presented by these varying vascular conditions, and the signaling mechanisms that trigger those phenotypes, we may gain information critical to growing our understanding of vascular biology and vascular development. While it may be challenging to examine these variables, we have several currently available cellular and animal models which can be exploited to decipher these questions. Indeed, recent advances in proteomics and transcriptomics are likely to enable such a fine-grained analysis that may point to subtle differences that cumulatively lead to larger differences in vessel function.

### Beyond the endothelium: immune cells

While circulating immune cells are not classically considered part of the cardiovascular system, they do directly signal to the endothelium and thus can impact the local vessel environment. Both human CCM samples and mouse CCM models frequently exhibit immune cell infiltration, including plasma cells, B-cells, T-cells, neutrophils, and macrophages [85]. Yau et al. recently reported that in an endothelial-specific *PDCD10* knockout mouse model, the presence of neutrophils increases in CCM lesions over time. Furthermore, an increased frequency of neutrophil extracellular traps is seen in mature lesions, particularly in the presence of a thrombus [86], suggesting that wild-type neutrophils are responsive to local lesion conditions. On the other hand, infiltration of these cells is not more prevalent in lesions with recent growth or hemorrhage [85], suggesting that immune cell infiltration may not be solely an acute response to injury. Regardless, immune cells could contribute to the pathogenesis of CCM through direct or indirect signaling to the endothelium, consequently promoting an inflammatory milieu within the lesion.

Several studies support the idea that immune cells modify lesion phenotype. For example, polymorphisms in TLR4 (a widely expressed LPS receptor), CD14 (monocyte/macrophage marker), and IL-6R (leucocyte cytokine receptor) are associated with the presence of intracerebral hemorrhage in CCM [87]. TLR4+ and TLR2+ T-cells (CD4+ and CD8+) are enriched in the peripheral blood of symptomatic CCM patients as are Th-17-like cells. In addition, these T-cells are more sensitive to LPS, suggesting that symptomatic CCM patients have a more pro-inflammatory T-cell phenotype [88]. However, these studies do not address whether local and systemic immune responses exert differential effects on CCM phenotype.

The first evidence that the presence of local immune cells directly affects CCM pathogenesis followed from the discovery that plasma cells found in CCM lesions produce oligoclonal IgG proteins [85], akin to autoimmune responses seen in multiple sclerosis and Kawasaki disease. The antibodies produced by these oligoclonal cells bind to both endothelial cells and astrocytes within the CCM lesions and associate with cytoskeletal proteins in these cell types [89]. Notably, these antibodies are not found in serum from the same patients [85]. Systemic B-cell depletion using an anti-BR3 antibody in a ‘sensitized’ PDCD10 mouse model (*PDCD10^+/−^ Trp53^−/−^* mice) was sufficient to decrease total lesion number, lesion area, and local non-heme iron staining (a marker of lesion bleeding). This effect was most pronounced in larger and more complex lesions [90], suggesting that B-cell depletion affected lesion growth and progression. However, whether the decrease in lesion burden is due to the absence of oligoclonal IgG proteins in the lesion, or some other B-cell dependent effect, is unclear. Nevertheless, this study, in which the disease-causing mutation is present in all cells, not just endothelial cells, supports the idea that loss-of-function mutations in CCM genes can directly alter the function of immune cells. This possibility was first raised by our discovery that adoptive transfer of wild-type bone marrow into KRIT1 heterozygous mice (*Krit1^+/−^*) restored vascular permeability in KRIT1 deficient mice to control levels [91]. As increased permeability is tightly linked to CCM development, severity, and progression, these data suggest that loss of KRIT1 expression in bone marrow–derived cells may play a role in endothelial dysfunction and CCM disease pathogenesis.

As a proof of concept, we recently demonstrated that Cre-dependent deletion of KRIT1 in neutrophils caused reduced adhesion to fibronectin, increased migration, and decreased integrin activation *in vitro*, and decreased neutrophil infiltration in a mouse model of acute respiratory distress [92]. While we are still investigating the molecular mechanism(s) involved, several ways KRIT1 could regulate neutrophil adhesion are supported by the literature. For example, KRIT1 binds to the small GTPase Rap1, an integrin regulator involved in the recruitment of talin-1 to the β integrin cytoplasmic tail [24,93,94]. Though we recently reported that Rap1 binding is not required for endothelial KRIT1 to maintain barrier function [15], we speculate that KRIT1 could modify Rap1-mediated integrin activation in neutrophils by competing with other Rap1 effectors for binding to Rap1. On the other hand, RhoA/ROCK activity is important for neutrophil function [95–99]; therefore, the possibility that KRIT1 expression could regulate RhoA in neutrophils is an attractive hypothesis. Indeed, our recent data suggest that KRIT1-deficient neutrophils exhibit increased baseline RhoA/ROCK activity [15]. Alternatively, KRIT1 could affect neutrophil adhesion via regulation of ICAP1α; however, the consequence of KRIT1 depletion on integrin regulation by ICAP1α is somewhat controversial, given that both positive and negative effects on integrin activity have been reported [13,14]. Nevertheless, as ICAP1α is an important regulator of integrin activity and turnover [100], it is feasible that this pathway could underlie the effect of reduced KRIT1 expression in neutrophils. It remains to be seen whether loss of KRIT1 in neutrophils contributes directly to CCM lesion pathogenesis, nevertheless, the cumulative evidence strongly suggests that immune cells and the inflammatory response play a major role in CCM pathogenesis and progression. While more work to delineate this role is needed, this mechanism may be a prime target for future therapeutics.

## KRIT1: beyond vascular biology

### KRIT1 in extra-vascular tissues

**Figure 3:**
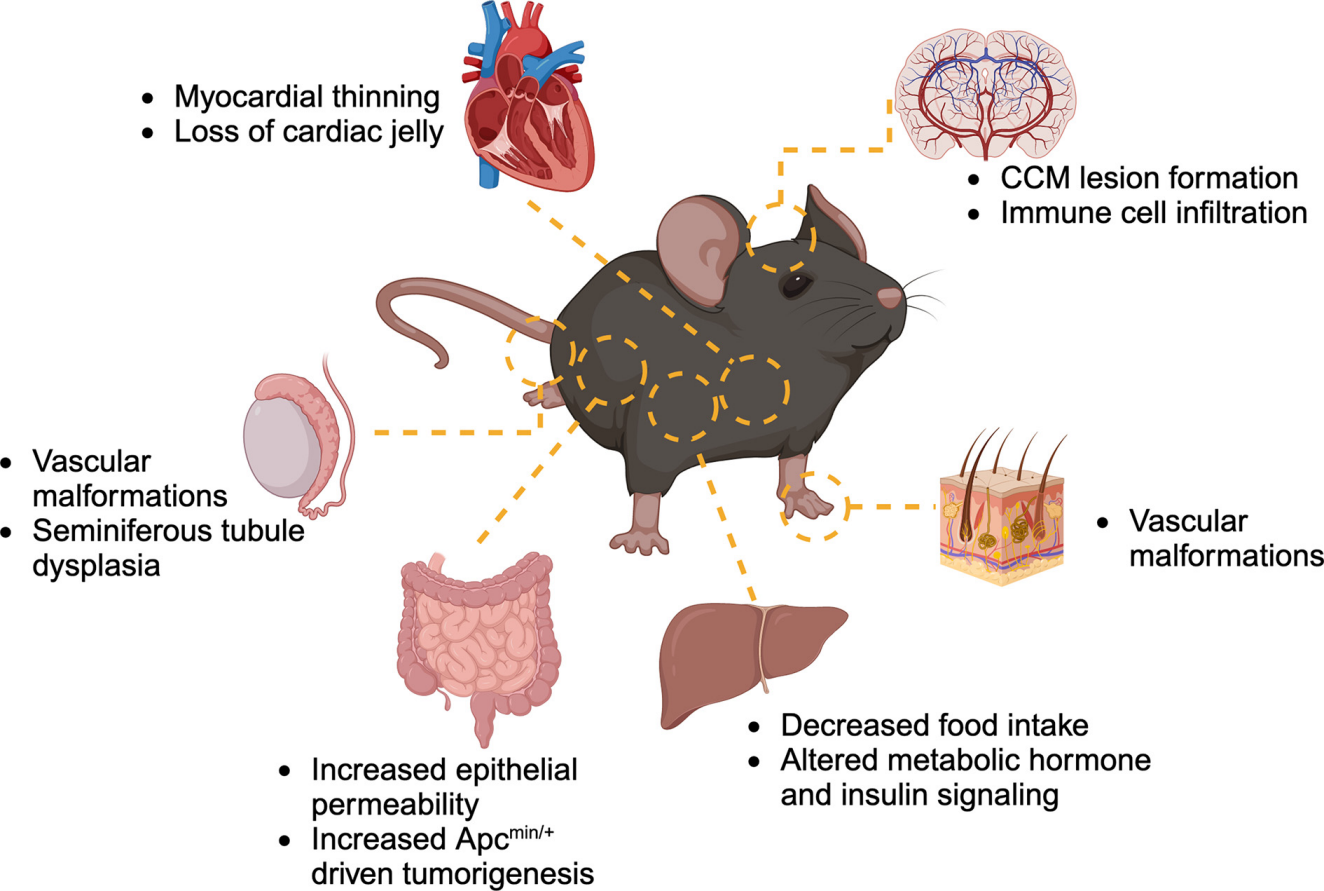
Tissues affected by loss of KRIT1 Clockwise from upper right, loss of KRIT1 expression in endothelial cells in the brain leads to CCM lesion formation with accompanying immune cell infiltration. Vascular (capillary) malformations are observed in the skin in 3–5% of CCM patients. Krit1 heterozygous mice exhibit decreased food intake and altered metabolic signaling linked to reduced KRIT1 expression in the liver. Loss of KRIT1 expression in the intestinal epithelium potentiates tumor formation in the presence of an oncogene and increases intestinal epithelial permeability. Deletion of KRIT1 in endothelial cells causes vascular malformations in the testis. KRIT1 heterozygous mice also exhibit seminal vesicle dysplasia. In the heart, loss of KRIT1 protein leads to myocardial thinning due to degradation of cardiac jelly by ADAMTS4 and ADAMTS5. Created with Biorender.com.

KRIT1 is ubiquitously expressed, and in fact is expressed in some cell types at much higher levels than in endothelial cells [25,92]. Recently, more attention has been paid to the function of KRIT1 in non-hematopoietic cells and tissues, including the heart, liver, and gut (Figure 3). In the heart, deletion of KRIT1 in the endocardium led to thinning of the myocardium and loss of cardiac jelly, due to increased expression of *Adamts4* and *Adamts5* proteases [101]. This mechanism also likely explains the phenotype conferred by loss of KRIT1 signaling in zebrafish embryos; a dilated, thin-chambered heart [102]. In the liver, heterozygous loss of KRIT1 was associated with decreased food intake and changes in metabolic hormones, as well altered insulin signaling [103]. In the mammalian gut, KRIT1 is expressed in the small intestine and colonic epithelium [104]. However, the first report of KRIT1 in the gut was in *Caenorhabditis elegans*, where *kri-1*, the worm orthologue of KRIT1, is involved in signaling between the reproductive system and the intestine that mediates the extension of life span following germ cell removal [105]. Later, it was shown that knockdown of *kri-1* decreased germ cell death in a cell non-autonomous fashion through decreased expression of the zinc transporter zipt-2.3 [106,107], suggesting that one function of KRIT1 in the gut is zinc homeostasis.

While the exact mechanism(s) of KRIT1 signaling in non-endothelial cells are not fully defined, some similarities to KRIT1 signaling in endothelial cells have been observed. KRIT1 appears to regulate epithelial cell–cell contacts in the same way it regulates adherens junctions in endothelial cells. Loss of KRIT1 in MDCK cells causes dissociation of β-catenin from the adherens junction, and enhanced β-catenin transcriptional activity that leads to cell-intrinsic changes in gene expression [25]. In addition, Wang et al. recently observed that stable knockdown of KRIT1 in Caco-2 intestinal epithelial cells increased the permeability of the epithelial monolayer and reduced claudin-1 expression, but unlike in endothelial cells, failed to stimulate actin cytoskeletal remodeling. KRIT1 deficient Caco-2 cells were also more sensitive to increased permeability and apoptosis stimulated by TNF-α [104], which has also been observed in KRIT1-deficient endothelial cells [91]. Interestingly, KRIT1 deletion in the intestinal epithelium *in vivo* did not exacerbate CCM-like lesion formation in KRIT1 endothelial knockout mice [104], in contrast with epithelial deletion of PDCD10 (in PDCD10 endothelial knockout mice), which increased lesion formation and reduced the thickness of the colonic mucosal barrier [108]. This may indicate that the different CCM proteins have divergent roles in non-endothelial cells.

The few studies published to date in non-vascular cells and animal models have likely only scratched the surface of the processes and behaviors in which KRIT1 signaling is involved. A trawl through the Gene Expression Omnibus (GEO) repository (https://www.ncbi.nlm.nih.gov/geo/) returns 55 transcriptomic studies in which KRIT1 was significantly differentially expressed, including in Duchenne muscular dystrophy (up-regulated) [109], tibial muscular dystrophy (down-regulated) [110], severe Alzheimer’s disease (down-regulated) [111], and severe acute respiratory syndrome (SARS, down-regulated) [112]. Thus, we may yet discover new KRIT-1 dependent mechanisms in other cell and tissue types, and conversely, these discoveries may fuel a better understanding of CCM.

### KRIT1 as a tumor suppressor

It has been proposed that KRIT1 acts as a tumor suppressor multiple times over the past two decades. This is due, in part, to the development of a model of CCM pathogenesis which follows Knudsen’s two-hit theory of tumor suppressors. Knudsen’s theory holds that tumors can arise from two inactivating mutations targeting both alleles of a putative tumor suppressor gene [113]. In CCM, several studies have supported the hypothesis that CCM lesion formation requires the acquisition of biallelic loss-of-function mutations in a CCM gene. In patient samples, lesional endothelial cells have been shown to exhibit biallelic mutations in CCM genes, albeit with low frequency [42]. In CCM gene knockout mouse models, mutations in both alleles are also required for lesion genesis, as heterozygous mice fail to form lesions [29,30]. However, crossing heterozygous *Krit1* animals (*Krit1^+/−^*) with mice lacking the tumor suppressor *Trp53* (p53) [114] or the mismatch repair gene *Msh2* [115] demonstrated that increased rates of somatic mutation are sufficient to promote lesion genesis in heterozygous animals, supporting the two-hit hypothesis.

More recently, advances in sequencing technology and the discovery that a high proportion of CCM patients also carry activating mutations in *PIK3CA* [34,116] have revised the two-hit hypothesis into a three-hit hypothesis. This model proposes that biallelic inactivation of a CCM gene plus an activating mutation in an oncogene such as *PIK3CA* or *Akt1* leads to lesion development and progression. In animal models which have biallelic loss of a CCM gene in the absence of an oncogenic mutation, it is proposed that an additional growth stimulus is required (i.e. increased VEGF expression [26]) to stimulate lesion development, which may explain why knockout of these genes must be induced within a specific developmental period to yield lesion-bearing mice [117]. Furthermore, using the *Cdh5* (PAC)-CreERT2/R26R-Confetti mouse that carries the stochastic multicolor reporter Brainbow2.1 in the R26 locus, Detter et al. [118] and Malinverno et al. [119] determined that CCMs arise via clonal expansion of a single mutated endothelial cell, which as lesions progress, recruits neighboring wild-type endothelial cells into the growing lesion. Malinverno et al. went on to show that this recruitment induces endothelial-to-mesenchymal transition in the wild-type cells [119], which had already been reported as a characteristic of CCM in human samples and animal models. Based on this evidence, CCMs could be considered endothelial cancers, or hemangiomas, where the CCM proteins would fill the role of tumor suppressor.

Beyond the endothelium, the concept of CCM proteins as tumor suppressors is also well supported for epithelial and other cancers. First, we demonstrated that animals heterozygous for KRIT1 (in all cells) and bearing a loss-of-function mutation in the adenomatous polyposis coli gene (*Apc^Min/+^*) developed a greater number of intestinal adenomas and demonstrated increased nuclear β-catenin localization when compared with animals expressing only the mutation in *Apc*. *Krit1^+/−^ Apc^Min/+^* mice also exhibited reduced survival due to increased tumor burden [25]. KRIT1 is also the target of miR-21 [120], a microRNA over-expressed in many tumor types that displays oncogenic activity [121,122]. miR-21 mediated silencing of KRIT1 increases anchorage independent growth of MC-1 melanoma cells and MDA MB-231 breast cancer cells. Indeed, specific shRNA-mediated knockdown of KRIT1 in these cell lines is sufficient to increase anchorage independent growth 2-fold, and conversely, over-expression of KRIT1 reduced anchorage independent growth [120]. This is in agreement with an early paper, in which dibutryl cyclic adenosine monophosphate-mediated reverse transformation (mesenchymal-to-epithelial transition) was marked by a significant up-regulation of *Krit1* expression [123]. Ercoli et al. also reported that KRIT1 expression is decreased in dysplastic, growing, and metastatic melanomas compared with normal skin or a benign common nevus. KRIT1 deletion in A375 melanoma cells led to increased proliferation, migration, and invasion, increased localization of β-catenin in the nucleus, and increased expression of markers of epithelial-to-mesenchymal transition [124]. Finally, a recent study reports that expression of *Krit1*, *Ccm2*, and *Pdcd10* are reduced in metastatic breast cancer tissues compared with the primary tumor [125], which indicates that CCM proteins might participate in tumorigenesis and metastasis.

Despite this evidence, the labeling of KRIT1 as a tumor suppressor gene remains controversial. Some studies have found higher relative RNA expression levels of KRIT1 and CCM2 in cancer cell lines, as compared with normal primary cell lines [39,40], which would not be expected of a tumor suppressor. In addition, some have pointed to a lack of association of KRIT1 with cancer in genome wide association studies. However, the NCI Cancer Genome Atlas Program (https://www.cancer.gov/ccg/research/genome-sequencing/tcga) currently contains >30 projects reporting cancer associated mutations or copy number variations in KRIT1 using a variety of approaches, including RNAseq, whole genome sequencing, whole exome sequencing, proteomics, and more. Thus, as more and more evidence accumulates which supports the classification of KRIT1 as a tumor suppressor, we also continue to learn more about the role of KRIT1 in cancer biology, which may yield surprising insights into the function of these proteins in vascular biology, and beyond.

## Caveats and closing remarks

In this review, we have discussed the contribution of KRIT1 to blood vessel morphology, barrier regulation, immune cell function, cancer biology, and examined what is known about the role of KRIT1 in non-vascular cell types. However, all these functions are also attributed to the other CCM proteins, CCM2 and PDCD10. Thus, while we have, wherever possible, limited the discussion to studies involving KRIT1; in several cases, this has not been possible as the relevant studies have only been performed in the context of CCM2, or more commonly, PDCD10, deletion. This highlights an important caveat, which is that it is commonly assumed or implied that all three CCM proteins behave equivalently in every condition. This is a dangerous assumption, as we know that patients with PDCD10 mutations tend to have a more severe phenotype and earlier onset [126], suggesting at least some variation in the disease process. In addition, subtle differences in how the animal models are implemented, such as timing, amount, or route of tamoxifen administration, can have large effects on study outcome, making it difficult to compare results between studies. Furthermore, acute studies, in which lesion formation occurs rapidly and the animals typically do not survive past P30, fail to recapitulate the progression of the human disease, and though they do afford a more rapid study timeline, they may not accurately reflect the physiology of lesion development.

The preceding statement that acute studies ‘may not accurately reflect the physiology of lesion development’ highlights another important caveat to this discussion, which is that we don't fully understand the natural history of CCM development, particularly in human patients. This lack of knowledge affects how we think about changes in vascular morphology driven by the CCM proteins, how immune cells contribute to CCM formation or progression, and how components of the neurovascular unit are affected by the developing CCM. Do lesions form through remodeling of existing tubes or do they grow like sprouts from normal vessels? Are immune cells recruited to CCMs at a specific point of lesion development? At what stage does astrocytosis begin, or at what stage do astrocytes, pericytes, and neurons dissociate from the vessel wall? We can begin to answer these questions using existing animal models; however, most studies to date have only examined one or two timepoints as snapshots of early or late lesion development. Introduction of longitudinal techniques, like repeated MRI or cranial window imaging, or even just expanding the timeline of traditional approaches can increase the temporal resolution of our knowledge and allow us to answer these questions and more. Unfortunately, the rarity of the disease limits the availability of human CCM samples, and the fact that only clinically active lesions are removed surgically likely biases the phenotype of samples we do obtain.

A final caveat to consider is the heterogeneity of CCMs. CCMs present clinically with significant heterogeneity in disease severity, symptoms, and anatomical location, even among family members who possess the same genetic mutations. The disease is also incompletely penetrant, meaning that some genetic carriers never develop lesions [43]. None of our current models replicate this heterogeneity well, by design. The animal models are designed to produce a uniform phenotype on which interventions can be tested. However, they often fail to even recapitulate the correct genotype of familial CCM patients, in which the lesion endothelium is negative for KRIT1/CCM2/PDCD10 expression and all other tissues are heterozygous, and instead model homozygous null endothelium on a wild-type tissue background. In order to understand the cause of heterogeneity in CCM (or cancer, etc.), new models will need to be developed. Take, for example, the ‘third hit’. In mice, homozygous deletion of KRIT1 is sufficient to cause lesion development, but only if the deletion occurs within a specific developmental window which coincides with blood vessel development in the brain [29,117]. However, local introduction of a gain-of-function mutation in *PIK3CA* is sufficient to form lesions in mice which were depleted of PDCD10 outside of that developmental window [34]. Despite the finding that many CCM patients also bear activating mutations in *PIK3CA*, it remains an open question whether an activating oncogenic mutation is required for lesion formation in human patients, or whether, as in mice, another growth stimulus could play the same role. The variation in the timing, gene identity, and penetrance of the three ‘hits’ likely play a significant role in the heterogeneity seen in CCM patients. In addition, there may be other, as yet undiscovered, genetic or environmental factors that modulate lesion development, clinical progression, and bleeding rates. The development of new models that successfully model this heterogeneity will be critical to mining this disease feature for therapeutic targets.

In conclusion, it seems we have yet to run out of important questions regarding the pathogenesis of CCM and the function of KRIT1. While challenging, tackling these questions is likely to improve not only our understanding of CCM pathogenesis, but to also address questions regarding basic biochemical mechanisms, such as the crosstalk between cell adhesion complexes and transcriptional activity. Recent evidence supports the hypotheses that KRIT1 regulates the function of both endothelial and non-endothelial cells and may play a role in cancer biology. Future studies can explore new signaling paradigms involving KRIT1 and the CCM proteins that could be exploited to provide new therapies for CCM, other cardiovascular diseases, or even cancer.

## Abbreviations

AVM: arteriovenous malformation
CM-AVM: capillary malformation-arteriovenous malformation syndrome
HHT: hereditary hemorrhagic telangiectasias
KTS: Klippel-Trénaunay syndrome
PWS: Parkes-Weber syndrome
SARS: severe acute respiratory syndrome
SWS: Sturge-Weber syndrome
VM: venous malformation
